# Dr. Mercier and Asylum Workers

**Published:** 1918-06-22

**Authors:** 


### DR. MERCIER AND ASYLUM WORKERS.

### A MANSION HOUSE SPEECH AND SOME REMINISCENCES.

It is peculiarly appropriate that I should be called upon to support, in the historic Mansion House of the City of London, this resolution with respect to asylum workers, for there is only one thing with which I have been associated longer than with asylum work, and that one thing is the City of London. I went to school in the City of London, at the Merchant Taylors' School, which was then in Suffolk Lane, a stone's throw from this spot. The system of instruction there was by means of the cane. We were caned for everything, and we were caned for nothing. I have held out my hand and received six tremendous strokes with the cane upon it, so that it was numb, and paralysed, and swollen, and then have been sent back to write a copy; and because my copy was badly written I have been caned again. It was a doleful experience; but I would rather go back to school and be caned again than be a nurse to mad people. I started on the voyage of life from St. Katharine's Dock in the City of London, as cabin-boy on board a small brigantine. The life was hard. We took our meals in this fashion: a lump of meat was put in a wooden trough and placed on the deck before us. We sat round upon our sea-chests, with a knife in one hand and a biscuit in the other; we hacked pieces off the lump of meat, and ate them as best we could. Plates and forks were as unknown to us as champagne glasses and asparagus tongs. I took my clothes off once in six weeks, but whether the other members of the crew ever changed their clothes I do not know. The life was hard; but I would rather be a cabin-boy on board a coasting brigantine than be a nurse to mad people. My next venture also was in the City of London, in one of the streets leading off Cheapside. There I was employed in a warehouse. The hours were from eight o'clock in the morning until the work was done, which was sometimes at six o'clock at night, sometimes not until eight o'clock, or ten o'clock, or midnight, and sometimes not until the small hours of the morning. There was not much time for recreation; but I would rather be a warehouseman, working these long hours, than I would be a nurse to mad people. I was then employed, not in the City of London, but just outside it, in the shadow of Temple Bar, which was then still standing, and from which the heads of the traitors had been removed not so very long before. Again my hours were long, but at the other end of the day. On ordinary week-days I was on duty at a quarter to five in the morning, on Saturdays at a quarter to three; and part of my duty was to carry the sacks on my back to the carts waiting in the Strand to carry the goods to the early trains; and I would rather return and carry sacks at these hours than I would be a nurse to mad people. And I will tell you why.

My late friend, Sir Thomas Clouston, who was at the head of the great asylum in Edinburgh, to which doctors came from all parts of the world to learn how mad people ought to be treated, used to say that the insane were unloveable. If he had said all that was in his mind, and all that the facts justified, he would have said that they are detestable. Many mad people are physically filthy, so filthy as to outrage every sense, from the sense of decency to the sense of smell. The nurse has to clean them, and to keep them clean. Many mad people, whether they are physically filthy or not, are morally filthy. Their conversation, their demeanour, and their acts are such as to give one nausea to witness; and the nurse has to witness them, and to refuse to be contaminated by them. Many mad people are actuated by a spirit of malice which ranges from impish mischief to a deep and rooted malignity. I will give you an instance or two. At one time I was employed as medical officer in the asylum of the City of London, at Dartford—another association with the City of London. After I had left, I went back to re-visit the place and some of my former acquaintances. As I entered a ward, one of the patients rushed up to me and said "Hullo! they have let you out on bail, then!" An old gentleman under my care contrived to post some letters, which he was not permitted to post; he was very closely watched, and I asked him how he managed it. He said, "I left the letters on a seat by the roadside, and on them I placed a tobacco pouch full of tobacco, with a note explaining that whoever would post the letters was welcome to keep the tobacco pouch and its contents." "Well," I said, "you got your letters posted, but you lost your tobacco pouch: was it worth while?" "Why," he said, "do you suppose I was such a fool as to leave my own tobacco pouch?"

A third patient addressed me as follows: "I will murder you: I will have your life; and they cannot do anything to me, for I am a lunatic in an asylum. The worst that could happen to me would be to be sent to Broadmoor; and I would rather be there than here, for I am told they get more tobacco there."

A hundred years ago, the King who occupied the throne of these realms was mad. He was so distressed by the death of his favourite daughter, the Princess Amelia, that he lost his reason, and was placed in the care of two keepers, as nurses to mad people were called then. Thirty years ago I was acquainted with an old gentleman who had been born in the reign of George III., and he had known in early life one of these keepers who had charge of the King. My friend, Dr. Paul, asked the man what he did when the King became violent, and the answer was, "We knocked him down as flat as a flounder." That was the way in which the highest person in the realm was treated when he was mad a hundred years ago. If any nurse in the asylum

### DR. MERCIER AND ASYLUM WORKERS—(*Conclusion*).

of the City of London were now to treat in this way the meanest subject of King George V. he would be brought before you, my Lord Mayor, and you would send him to prison. He would lose his employment, he would lose his pension, and would be a ruined man. We are here to-day to do honour to asylum workers, who have carried on, under circumstances of unexampled difficulty, a most arduous and repulsive occupation. Before the war there were in the asylums of this country, 5,300 male nurses of military age. Of these men, four in every five have joined the Army. The efficiency with which asylums are conducted may be roughly estimated by the number of suicides that have taken place in them. In the asylums of England and Wales there were, in 1916, some 134,000 mad people, a very large proportion of whom were suicidally inclined, and a considerable portion were actively suicidal, and eager to take advantage of any opportunity for carrying out their purpose of self-destruction. In spite of this, and in spite of the depletion of the staffs, out of these 134,000 only four succeeded, in the year 1916, in carrying out their purpose. I need say no more, and I commend the resolution very heartily to your acceptance.

